# Research Progress on the Role of M6A in Regulating Economic Traits in Livestock

**DOI:** 10.3390/ijms25158365

**Published:** 2024-07-31

**Authors:** Tuanhui Ren, Meng Xu, Xinyu Du, Yanxi Wang, Juan J. Loor, Lin Lei, Wenwen Gao, Xiliang Du, Yuxiang Song, Guowen Liu, Xinwei Li

**Affiliations:** 1 State Key Laboratory for Diagnosis and Treatment of Severe Zoonotic Infectious Diseases, Key Laboratory for Zoonosis Research of the Ministry of Education, Institute of Zoonosis, College of Veterinary Medicine, Jilin University, Changchun 130062, China; tuanhuiren@163.com (T.R.); mengxu202312@163.com (M.X.); goodxyu@163.com (X.D.); ciciwang926@163.com (Y.W.); jiluleilin@126.com (L.L.); jilugaoww@126.com (W.G.); duxiliang@jlu.edu.cn (X.D.); songyuxiang2018@126.com (Y.S.); liuguowen2008@163.com (G.L.); 2 Mammalian NutriPhysioGenomics, Department of Animal Sciences and Division of Nutritional Sciences, University of Illinois, Urbana, IL 61801, USA; jloor@illinois.edu

**Keywords:** m6A methylation, methyltransferases, livestock, growth and development, reproductive traits

## Abstract

Reversible regulation of N6-methyladenosine (m6A) methylation of eukaryotic RNA via methyltransferases is an important epigenetic event affecting RNA metabolism. As such, m6A methylation plays crucial roles in regulating animal growth, development, reproduction, and disease progression. Herein, we review the latest research advancements in m6A methylation modifications and discuss regulatory aspects in the context of growth, development, and reproductive traits of livestock. New insights are highlighted and perspectives for the study of m6A methylation modifications in shaping economically important traits are discussed.

## 1. Introduction

Various types of modifications of eukaryotic RNA, the crucial intermediate in the process of gene expression, occur widely in nature. Currently, more than 160 types of RNA modifications have been discovered, with RNA methylation accounting for over 60% of these. Methylation modifications have distinct biological functions within cells [1]. RNA methylation refers to the chemical modification phenomenon in which a methyl group is selectively added to adenosine residues in RNA via methyltransferases to form primarily N6-methyladenosine (m6A). The presence of m6A in mammalian cells was first reported in 1974, but early research into its potential functions was limited due to the lack of technology for studying how these modifications affected cell function [2]. In 2011, the obesity-associated protein (FTO alpha-ketoglutarate dependent dioxygenase) was reported to effectively remove m6A modifications from RNA, suggesting that these modifications could have a regulatory role [3]. It was not until 2012 that mRNA transcripts corresponding to m6A peaks in mouse and human cells were first identified using MeRIP-Seq (also known as m6A-seq). A high level of conservation of these m6A peaks across species was detected [4,5]. The development of m6A-seq technology has, in fact, allowed the detection of m6A modifications in plants, animals, viruses, and other organisms [6,7,8,9,10].

Modifications induced via m6A methylation of adenosine residues on messenger RNA (mRNA) and non-coding RNA (ncRNA) are reversible and ubiquitous. They are dynamically regulated in a cell-specific manner via methyltransferases (writers), demethylases (erasers), and m6A-binding proteins (readers) [11]. By influencing mRNA splicing, translation, stability, export, RNA structural changes, and the maturation process of miRNA, methylation via m6A constitutes an important epigenetic control of cell function [2]. It plays a significant role in biological processes such as embryonic development, fat depot metabolism, muscle development, and disease onset and progression [10,12,13]. The role of m6A methylation in the occurrence and metastasis of cancer has received special attention in the human medicine field. For instance, in acute myeloid leukemia (AML) cell lines, the absence of the RNA m6A writer WTAP can inhibit proliferation and promote differentiation and apoptosis [14]; the deletion of the methyltransferase catalytic subunit METTL3 suppresses translation of its target genes (*MYC*, *BCL2*, and *PTEN*), thereby promoting differentiation and apoptosis of cancer cells [15]; METTL14 increases m6A methylation of *MYB* and *MYC*, inhibits the differentiation of AML cells, and promotes cell proliferation, leading to tumorigenesis [16].

In the context of cancer diagnosis, prognosis, and prediction, elucidating the mechanisms for dysregulation of RNA m6A modifications and its related mechanisms can serve to identify effective biomarkers [17]. This epigenetic modification also plays a crucial role in regulating plant growth, development, and stress resistance. For instance, ZmMTA is a key mRNA adenosine methylase in maize (the core component of the m6A methyltransferase complex) and its loss of function can severely impair endosperm development, underscoring the importance of m6A modifications [18]. In field trials, the transgenic expression of the RNA demethylase FTO led to an approximate 50% increase in the yield and biomass of rice and potatoes, and the presence of FTO stimulated proliferation of plant root meristematic cells along with enhanced photosynthetic efficiency and drought resistance [19].

Increased focus on genomic-related research has contributed to unraveling the genetic mechanisms underlying important economic traits in livestock, with the end goal of enhancing production efficiency, reproductive capacity, and product quality. In this review, we discuss recent advances in m6A methylation modifications and the regulatory roles of m6A during growth, development, and reproduction in livestock. Due to the vast number of publications in recent years on m6A, this review cannot cover all the available literature. We briefly list and summarize the key influential research findings on m6A methylation modifications in economically important traits of livestock. Lastly, we discuss the emerging challenges and outstanding issues in this field. To date, despite significant progress in the study of m6A methylation modifications in species such as humans and mice, research on its relevance to economically important traits in livestock remains in the exploratory stage, calling for further in-depth investigations. Thus, a comprehensive review of the relevant research on RNA methylation in livestock enables us to gain a deeper understanding of its potential role in production, providing a theoretical basis for further exploration.

## 2. Basic Regulatory Mechanisms of m6A

**Figure 1:**
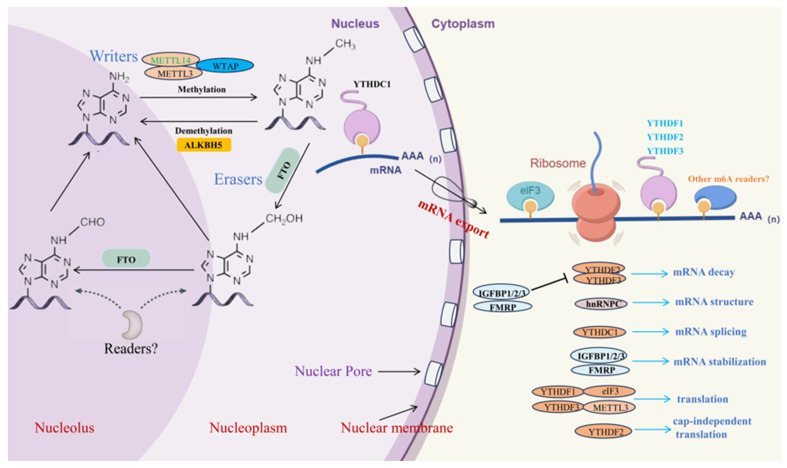
Dynamic process of m6A methylation modification (by Figdraw). The enzymes that write m6A modifications are protein complexes, which include METTL3, METTL14, WTAP, and other components. The FTO protein, a demethylase, removes the methylation from RNA by acting through its unique long loop region located at the C-terminal of its core domain. ALKBH5, another demethylase, demethylates RNA with m6A modifications through an oxidative process. Reader proteins such as YTHDF1, YTHDF2, YTHDF3, YTHDC1, and YTHDC2 are involved in various processes including mRNA splicing, stability, translation, and export.

m6A modifications are a common type of RNA modifications. This epigenetic modification alters the chemical properties and spatial structure of RNA molecules, thereby influencing their functionality [20]. The m6A modification is dynamically reversible and involves complex interactions among various enzymes, including methyltransferases (such as METTL3, METTL14, and WTAP), demethylases (such as FTO, ALKBH5, and ALKBH3), and m6A-binding proteins (such as YTHDF1, YTHDF2, YTHDF3, YTHDC1, and YTHDC2) [21,22,23]. Research indicates that m6A modification can affect mRNA splicing, stability, export, and translation [24,25], as well as the processing and function of miRNA [26] (Figure 1).

## 3. Function of m6A Modifications in Animal Growth and Development

### 3.1. Regulation of m6A Modifications in Muscle Development

**Figure 2:**
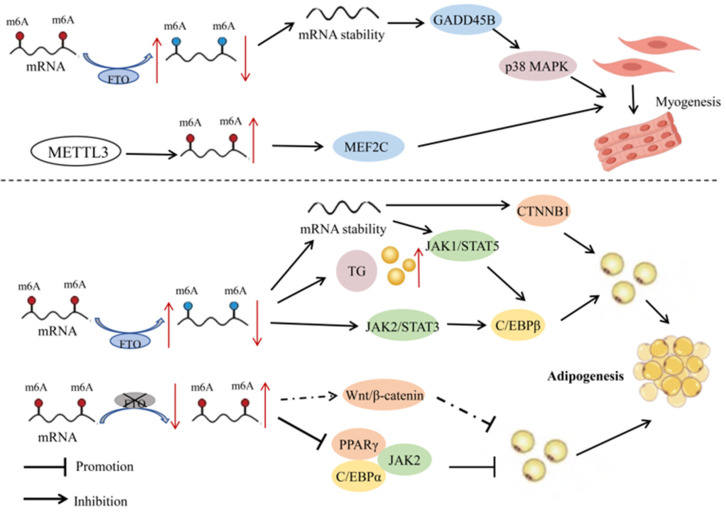
Mechanisms of RNA methylation in the development of muscle and fat in livestock. In myocytes, FTO increases the stability and expression of *GADD45B* mRNA by reducing the degree of m6A modification, thereby activating the p38MAPK pathway and promoting myoblast differentiation. METTL3, on the other hand, promotes the differentiation of goat skeletal muscle satellite cells by upregulating m6A levels and enhancing the expression of *MEF2C*. In adipocytes, FTO modulates the JAK2-STAT3-C/EBPβ signaling pathway through demethylation, which promotes the differentiation of porcine preadipocytes. The overexpression of FTO can also significantly increase the content of triacylglycerol (TG) in cells. Furthermore, FTO promotes the proliferation and differentiation of chicken preadipocytes by regulating the expression of *CTNNB1*. Silencing FTO significantly reduces the expression of *PPARγ* and *C/EBPα*, inhibiting the proliferation of porcine intramuscular preadipocytes. It may also be involved in the differentiation of porcine intramuscular preadipocytes through the Wnt/β-catenin pathway. In porcine BMSCs, the absence of METTL3 increases the stability of *JAK1* mRNA, activating *C/EBPβ* through the JAK1-STAT5 signaling pathway, thereby inducing the differentiation of BMSCs into adipocytes. Red and blue dots represent states of hypermethylation and hypomethylation, respectively. Dashed lines represent potential regulatory mechanisms. Symbols: ↑ indicates upregulation; ↓ indicates downregulation.

As the most abundant type of mRNA modification, m6A plays a regulatory role in skeletal muscle differentiation, myocardial remodeling, and regeneration [27]. The m6A modifications during six different stages of pig embryonic development revealed highly dynamic changes, with most of the affected genes enriched in pathways related to skeletal muscle development. The m6A methylation level of genes in porcine prenatal skeletal muscle significantly decreases at embryonic age E40 (the period of primary myofiber formation), and then gradually increases with the increase of embryonic age (E50–95 days) (the period of secondary myofiber formation) [27]. Silencing of IGF2BP1 by siRNA can promote proliferation and inhibit myoblast differentiation. The fact that skeletal muscle development is at the core of efficient meat production in livestock underscores the importance of m6A modifications on product quality. Research in goats has demonstrated that FTO can increase the stability and expression of *GADD45B* mRNA by reducing the degree of m6A modifications, thereby activating the p38MAPK pathway and promoting myoblast differentiation [28] (Figure 2). The protein METTL3 can also promote myogenic differentiation of goat skeletal muscle satellite cells by upregulating m6A levels and enhancing the expression of *MEF2C* [29]. These studies provide a potential molecular mechanism for the regulation of myogenesis in goats through RNA methylation.

In cattle, METTL3 (an m6A methyltransferase) has an inhibitory effect on the proliferation of myoblasts and can promote cell differentiation. It can also regulate myoblast development by affecting the stability of *TM4SF1*. The YTHDF2-mediated mRNA degradation of *TM4SF1* is also dependent on m6A modifications [30]. The expression of m6A demethylases (FTO and ALKBH5) in the longissimus of adult cattle is significantly higher than that in newborn cattle, while the expression of m6A methyltransferases (METTL3, METTL14, and WTAP) is lower. During myogenesis, the mRNA expression of these five genes is significantly increased; interference experiments in bovine myoblasts further indicated that m6A-methylation-related enzymes may regulate the development of bovine skeletal muscle [31].

In Jingxing-Huang chickens, the expression levels of m6A demethylase genes ALKBH5 and FTO increase significantly during skeletal muscle development. The proteins ALKBH5 and FTO may affect chicken skeletal muscle development by regulating RNA m6A methylation levels [32]. There is evidence that METTL3, METTL14, and WTAP have a regulatory role in chicken muscle fiber type composition, maintenance, and myoblast differentiation [33].

m6A is a key regulatory factor affecting the function of ncRNAs, thereby participating in the growth and development of organisms. Overexpression of METTL3 downregulated the expression of muscle-specific miR-1a, miR-133a, miR-133b, and miR-206 in differentiating C2C12 cells and a mouse model of muscle injury regeneration [34]. Researchers used RNA-seq and MeRIP-seq technologies to explore the m6A methylation patterns of lncRNAs in the oxidative and glycolytic skeletal muscles of pigs. The results showed that most lncRNAs have an m6A peak that is preferentially enriched in the last exon of the lncRNAs, and there is a positive correlation between the m6A levels and the expression levels of the lncRNAs. In addition, inhibition of lncRNA *MSTRG.14200.1* delayed the differentiation of satellite cells and stimulated the transition between fast and slow muscle fibers. This study provided new targets for the conversion of muscle fiber types in pigs [35]. In cattle–yak skeletal muscle, the abundance of m6A is positively correlated with gene expression levels but negatively correlated with lncRNA expression levels, suggesting that m6A modifications may play an important role in the development of cattle–yak skeletal muscle, but the regulatory mechanisms of m6A modification on mRNA and lncRNA may be different [36]. The use of RNA-seq and ATAC-seq sequencing technologies allowed the discovery of the skeletal muscle-specific lncRNA *MYH1G-AS*, whose transcription is coordinated by the transcription factors SMAD3 and SP2. In addition, SP2 inhibits the transcription of ALKBH5, weakening the demethylation of *MYH1G-AS* by ALKBH5, thereby disrupting the stability of *MYH1G-AS* RNA. *MYH1G-AS* accelerates the proliferation of myoblasts but inhibits their differentiation; this gene promotes the transition of slow muscle fibers to fast muscle fibers and leads to muscle atrophy [37]. Overall, these consistent results indicate that m6A methyltransferases play an important regulatory role in the development of skeletal muscle in livestock.

### 3.2. Regulation of m6A Modifications in Adipogenesis

There is evidence for m6A modifications in the regulation of expression levels of transcription factors and adipose tissue-specific genes, thereby influencing the process of fat depot metabolism. Among them, FTO, as an RNA demethylase, plays an important role in gene expression of the modified genes. For example, overexpression and specific knockout of FTO and METTL3 genes in porcine fat cells revealed a negative correlation between FTO and m6A methylation levels and a positive correlation between FTO and adipogenesis [38]. In contrast, METTL3 had a positive correlation with m6A methylation levels and a negative correlation with adipogenesis. In porcine bone-marrow-derived mesenchymal stem cells (BMSCs), the absence of METTL3 led to a decrease in m6A modification on *JAK1* mRNA, which in turn enhanced the stability and translational efficiency of *JAK1* mRNA. The JAK1-STAT5 signaling pathway is one of the key pathways regulating adipocyte differentiation, activating downstream genes related to adipogenesis such as *C/EBPβ* by phosphorylating STAT5 and promoting its entry into the nucleus, thereby directing the differentiation of BMSCs towards adipocytes [39]. The enzyme FTO, through m6A demethylation, promoted the differentiation of porcine preadipocytes by regulating the JAK2-STAT3-C/EBPβ signaling pathway, mitotic clonal expansion, and cellular autophagy pathways [40,41,42]. In porcine intramuscular preadipocytes, silencing FTO significantly reduced the level of phospho-histone H3 and inhibited the proliferation of intramuscular preadipocytes. Silencing FTO also led to downregulation of peroxisome proliferator-activated receptor γ (*PPARγ*) and CCAAT/enhancer-binding protein α (*C/EBPα*), and upregulation of β-catenin expression. In addition, LiCl, a specific activator of the Wnt/β-catenin signaling pathway, can attenuate FTO-induced upregulation of *PPARγ* and downregulation of β-catenin. In porcine adipocytes, overexpression of FTO significantly increased the intracellular content of triacylglycerol and markedly upregulated expression of the adipogenesis-related gene *C/EBPβ*, while downregulating the mRNA expression of the lipolytic genes *ATGL* and *HSL*; knockdown of FTO led to the opposite results, suggesting that this protein may promote lipid accumulation in porcine adipocytes [38]. In summary, silencing FTO reduces the proliferation and differentiation of porcine intramuscular preadipocytes, and may affect the differentiation of porcine intramuscular preadipocytes through the Wnt/β-catenin pathway [43].

In Chinese native chicken breeds, excessive accumulation of abdominal fat has always been a significant issue in poultry production. High-fat diets can lead to weight gain and excessive fat deposition, affecting genes and pathways involved in lipid metabolism and adipogenesis [44]. The m6A methylation of RNA is negatively correlated with adipogenesis in chicken preadipocytes. Overexpression of FTO significantly inhibited m6A levels and promoted proliferation and differentiation of chicken preadipocytes, while silencing FTO had the opposite results. The enzyme FTO regulates the expression of *CTNNB1* through demethylation, thereby promoting fat deposition in chickens (Figure 2) [45]. In addition, studies have reported that the m6A methyltransferases METTL3, METTL14, and WTAP, as well as m6A demethylases FTO and ALKBH5, are all significantly upregulated in adipose tissue of adult Qinchuan cattle compared with newborn cattle. Their temporal expression patterns during in vitro preadipocyte proliferation and differentiation also exhibited significant differences. These results suggested that m6A modifications may play an important role in fat deposition in cattle [46], and that m6A methyltransferases have a potential role in regulating proliferation and differentiation of bovine preadipocytes. 

Current research has demonstrated that many circular RNA (circRNA) molecules have multiple m6A modification sites. For example, a total of 12 candidate m6A-modified circRNAs (m6A-circRNAs) were identified in the longissimus dorsi muscle of Queshan black pigs and Large White pigs; these m6A-circRNAs can act as miRNA sponge molecules and regulate fat deposition by constructing a ceRNA regulatory network. Enrichment analysis revealed that the parent genes of m6A-circRNAs and their adsorbed miRNA target genes are involved in pathways related to fat deposition, cell proliferation, and differentiation, suggesting that m6A-circRNAs may be particularly important in dictating pork quality [47]. These consistent findings indicate that m6A methyltransferases play a crucial role in fat deposition in cattle, pigs, and poultry.

## 4. The Functional Role of m6A Modifications in Reproductive Traits

### 4.1. Regulation of Spermatogenesis by m6A Modifications 

Spermatogenesis is a highly complex and specialized cellular developmental process that involves three stages: spermatogonial proliferation, meiosis of spermatocytes, and spermiogenesis [13]. Gene expression during spermatogenesis is tightly regulated at the transcriptional, post-transcriptional, and translational levels to ensure the accurate expression of stage-specific genes. Previous research using conditional gene knockout mouse models of the m6A RNA methyltransferases METTL3 and/or METTL14 revealed that the absence of METTL3 or METTL14 in early spermatogenic cells led to the loss of spermatogonial stem cells. However, only the simultaneous knockout of METTL3 and METTL14 in relatively later-stage spermatogenic cells (type A1 spermatogonia) resulted in impaired sperm formation. In humans, the expression of WTAP and ALKBH5 decreased during the process of spermatogenesis [48]; FTO and ALKBH5 are highly expressed in the testes of mammals, and genetic mutations in human FTO are closely related to a decline in semen quality [49]. Studies have reported that male mice with ALKBH5 gene defects had reduced fertility due to apoptosis affecting spermatogonial cells at the meiotic metaphase [50]. ALKBH5 is upregulated in the developing goat testes, and overexpression of ALKBH5 in goat spermatogonial stem cells promoted the transition from the G1 phase to the S phase and inhibited apoptosis [51]. Researchers have used m6A-seq, RNA-Seq, and ribosome profiling techniques to generate dynamic profiles of m6A RNA modification during different developmental stages of spermatogenesis. They reported that inactivation of m6A methyltransferases led to a decrease in m6A RNA modification levels, resulting in significant changes in the transcriptional and translational efficiency of genes involved in spermatogonial fate determination and sperm formation [52].

The mark of transcription elongation H3K36me3 (trimethylation of histone H3 at lysine 36) guides the co-transcriptional process of m6A modifications [51]. H3K36me3 interacts with METTL3 and METTL14 of the m6A methyltransferase complex, promoting m6A modification of nascent RNA. This crosstalk between histone and RNA modifications provides new concepts for the biogenesis of mRNA-m6A [51]. Although DNA methylation and m6A modifications are independent processes, they may jointly influence the regulation of gene expression and spermatogenesis. Non-coding RNAs, as epigenetic regulators, play an important role in the process of spermatogenesis. Among them, miRNAs, circRNAs, and PiRNAs regulate the normal development of male germ cells at the transcriptional, post-transcriptional, and translational levels, and may interact with m6A modifications. Previous research demonstrated that in mouse GC-1 spermatogonia cells, after METTL3 was knocked down, the construction of a circRNA–miRNA–mRNA regulatory network revealed that *H2afx* and *Dnmt3a* are hub genes in sperm meiosis. This study may provide new insights and potential therapeutic targets for the pathogenesis of abnormal spermatogenesis caused by abnormal m6A modifications [53].

**Figure 3:**
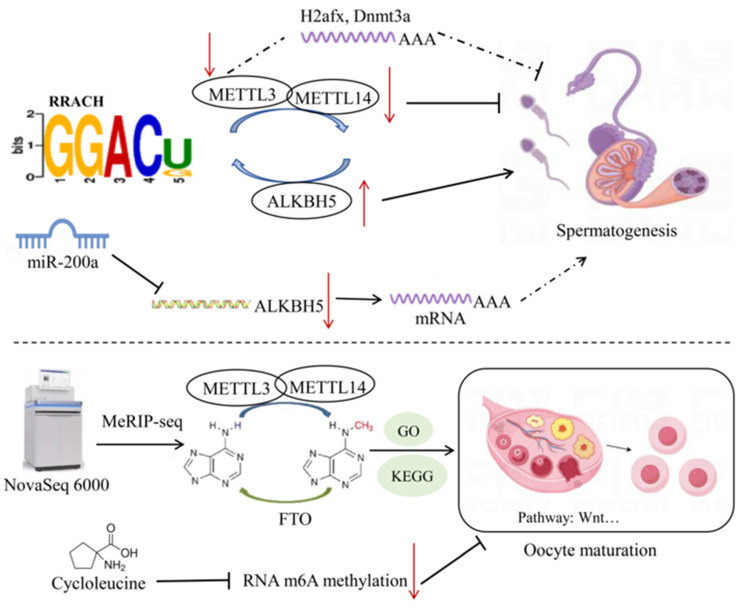
Mechanisms of RNA methylation in reproductive traits of livestock. After knocking down METTL3 in mouse GC-1 spermatogonial cells, PPI network analysis revealed that the core genes *H2afx* and *Dnmt3a* may play a role in spermatogenesis. In bovine testicular tissue, bta-miR-200a inhibits the expression of ALKBH5, thus affecting the regulation of spermatogenesis-related genes by m6A methylation. In porcine oocytes, cycloleucine inhibits m6A methylation, thereby hindering meiosis and early embryonic development. The dashed arrows in the figure represent mechanisms that are not yet clearly defined. Symbols: ↑ indicates upregulation; ↓ indicates downregulation.

All mRNA transcripts in pig germ cells contain m6A modifications, and the m6A reading density increases in the coding sequence (CDS) and reaches its highest value near the stop codon, then decreases in the 3′UTR [54]. During spermatogenesis, the abundance of m6A modifications in transcriptional products varies with different developmental stages. It was reported that the m6A modification reading density in the CDS was higher in pachytene spermatocytes and round spermatids, while it was higher in the 30 non-coding regions in spermatogonia [52]. Lack of METTL3 leads to abnormal initiation of spermatogonial differentiation and disrupts the ability of spermatocytes to reach the pachytene stage before meiosis [55]. In pachytene spermatocytes, upregulated methylated genes are preferentially involved in spermatogenesis and cell cycle processes, which are also upregulated by m6A modifications, indicating an important role of m6A in the early developmental stages. Furthermore, m6A modification in PS is enriched in genes involved in sperm cells development and upregulates the expression of genes involved in microtubule processing, cilia organization, and cilium assembly, suggesting that m6A-methylated mRNAs may inhibit translation before spermatogenesis [52]. In round spermatids, m6A-methylated genes are upregulated and regulated developmental processes including anatomical structure and tubular morphogenesis of multicellular organisms, indicating a conserved role of m6A modifications in mediating pig spermatogenesis [52]. During male germ cell development, the m6A RNA modifications are conserved and dynamically regulated, and are widely present in various stages of spermatogenesis, particularly highly enriched in the pachytene/diplotene spermatocytes and round spermatids [13]. In cattle, yak, and yak–cattle hybrid testicular tissues, bta-miR-200a regulated the m6A methylation levels by suppressing the expression of ALKBH5, affecting the expression and function of m6A target genes [56] (Figure 3). These consistent results suggest that m6A methyltransferases may be involved in animal spermatogenesis.

### 4.2. Regulation of Animal Oocyte Maturation via m6A Modifications

Oocyte maturation in animals can be divided into three main stages: meiotic maturation, cytoplasmic maturation, and epigenetic maturation [57]. Epigenetic maturation of oocytes primarily involves DNA methylation and histone modifications that regulate gene expression. In contrast, m6A methylation modifications can affect mRNA degradation rates and transcript length, and impede normal mRNA translation. This can lead to defects in follicle development, inhibition of oocyte maturation, early developmental arrest of oocytes, and impaired activation of the zygotic genome, thereby influencing and regulating various stages of oogenesis in female mammals [58].

The developmental competence of oocytes to support embryonic development is acquired during folliculogenesis, and interactions with surrounding granulosa cells during follicle development can affect oocyte quality [59,60]. Pig granulosa cells exhibit abundant m6A gene modifications, and dynamic changes in m6A methylation occur during the transition from small follicles (<3 mm) to large follicles (>5 mm). There are 7289 and 6882 m6A sites identified in granulosa cells from small and large follicles, respectively, with m6A being enriched near the 5′ or 3′UTRs and sharing conserved motifs. Further analysis revealed that differentially expressed m6A-methylated genes were significantly enriched in several signaling pathways related to steroidogenesis, granulosa cell proliferation, and follicle development [61]. Overall, these results highlight the presence of distinct m6A modifications in granulosa cells during pig follicle development, which may be associated with steroids and folliculogenesis.

The estrus cycle in yaks is a seasonal phenomenon that may involve the synthesis and secretion of sex hormones as well as epigenetic regulation of follicle growth and development [62]. HE staining revealed a significantly higher number of growing follicles and mature follicles in the ovaries during the estrus phase compared with the diestrus and pregnancy phases. qPCR results revealed significant differences in the expression of METTL3, METTL14, FTO, and YTHDC1 at different stages of ovarian development, suggesting a regulatory role of m6A modifications in ovarian activity. Additionally, the yak m6A transcriptome profile obtained through MERIP-SEQ technology revealed a high enrichment of m6A peaks in the CDS, 3′UTR, and conserved sequence “DRACH”. Results from GO, KEGG, and GSEA analyses indicated that m6A modifications may participate in many physiological activities of yak ovaries during the reproductive cycle [63].

In studies with poultry, somatic cells in the follicles determine the follicle selection process [64]. Analysis of m6A methylation of transcript levels during the follicle selection process in chickens revealed widespread mRNA methylation and demethylation in granulosa cells and follicular membrane cells, with m6A methylation modifications having a negative correlation with gene expression levels [65]. Both the peak m6A methylation and the modified transcripts increased during the follicle selection process, leading to dynamic expression of many genes related to folliculogenesis. Functional enrichment analysis suggested that m6A modifications of key factors in the Wnt pathway may play a major role in regulating follicle selection in chickens [65] (Figure 3).

Previous studies have revealed that treatment of oocytes with the m6A inhibitor (cycloleucine) blocked RNA m6A methylation and histone modifications, and altered the expression of metabolic genes during the blastocyst stage, thereby hindering meiosis and early embryonic development in pig oocytes [66]. These consistent results suggest that m6A methyltransferases may be involved in the maturation of oocytes in major livestock species.

## 5. Summary and Perspectives

The m6A modifications, widely present and an important type of epigenetic modification, play significant roles in various life processes of agricultural animals, including growth and development, lipid metabolism, and reproduction (Table 1). However, the functional impact of m6A modifications on mRNA is diverse and multifaceted, and its regulatory network is extremely complex. The dynamic processes of methylation and demethylation are regulated by multiple factors. Thus, in order to improve our understanding of the regulatory mechanisms and functional relationships of m6A modifications, it is necessary to conduct comprehensive evaluations and discussions based on specific biological processes and integrate findings from previous similar studies.

In recent years, integrative analysis of multi-omics has become one of the most important research methods in the field of life sciences. There is an increasing amount of research focusing on the combined analysis of m6A modifications with other omics. For example, the correlation between the transcriptome and m6A can provide a comprehensive explanation of the regulatory effects of transcription factors on gene expression and phenotypic differences. In the study of endometrial cancer, through MeRIP-seq, RNA-seq sequencing, and experimental analysis, it was discovered that IGF2BP1 recognizes m6A methylation sites near the stop codon of the target gene *PEG10*, thereby regulating endometrial cancer cell proliferation [91]. Furthermore, utilizing the pan-genome, RNA-seq, and functional studies, it was revealed that the deletion in the promoter region of the IGF2BP1 gene is likely the causal variant for the quantitative trait loci associated with chicken body size [92].

The m6A modifications are the most abundant methylation modification on mRNA, and are also present in long non-coding RNAs (lncRNA), miRNA, and circular RNA (circRNA). Previous studies have revealed that the m6A modification of the lncRNA *Dubr* stabilizes the YTHDF1/3 complex and regulates axon growth and neuronal migration through mRNA translation [93]. The m6A-modified *circNSUN2* can bind to YTHDC1 and promote its nuclear export. It further combines with IGF2BP2 to stabilize *HMGA2* mRNA, thereby promoting liver metastasis in colorectal cancer [94]. The m6A modifications during the development of mouse male germ cells can facilitate the generation of circular RNAs carrying open reading frames [95]. These findings contribute to understanding the regulatory mechanisms and functions of m6A-modified non-coding RNAs in various physiological or disease states.

Previous research indicated that the gut microbiota can regulate host DNA methylation and histone modifications [96]. In the process of gut microbiota–host interaction, modifications on host RNA, such as m6A, can also be influenced by microorganisms. The use of high-resolution mass spectrometry and m6A-seq RNA sequencing analysis revealed differences in m6A modification levels in multiple organs and tissues between germ-free mice and normal mice [97]. Under the influence of F. nucleatum, METTL3 can inhibit colorectal cancer metastasis by downregulating the expression of the target gene *KIF26B* [98]. The m6A modifications act as an important “information molecule” in the interaction between the host and microorganisms. The gut microbiota can regulate gene expression by altering host m6A RNA methylation modifications, and can also modify their own m6A modifications using host methyltransferases or demethylases. Correlation analysis between m6A modifications and omics data such as transcriptome, translatome, and microbiome is of great significance for exploring the specific mechanisms by which m6A modifications affect gene function. It helps enhance the depth and breadth of data analysis and provides a complete description of the transcriptional regulatory mechanisms. Thus, the combined analysis of m6A and other omics will be a key focus in future studies of economically important traits in livestock.

Compared with the research on m6A modifications in humans and mice, there is relatively little research on m6A modifications in livestock. Currently, studies using m6A-seq to evaluate the involvement of m6A modifications in economically important traits of livestock have mainly focused on methyltransferases, m6A levels, and the identification of differentially methylated genes related to traits. Thus, further research is needed to explore the specific functions and regulatory mechanisms of RNA-methylation-related enzymes and methylated genes in livestock. The study of RNA methylation still faces many challenges, such as potential false-positive results due to limitations in m6A-seq sequencing technology, and a lack of research on the dynamic nature of RNA methylation and regulatory mechanisms in livestock. We believe that with the improvement of RNA methylation databases and the development of RNA methylation detection techniques, especially the application of single-cell spatial transcriptomics and the emergence of more sensitive detection instruments, research on RNA methylation in livestock will become more comprehensive. More RNA methylation markers will be discovered and applied in livestock production processes, leading to greater breakthroughs in the study of m6A modifications.

The m6A modifications are commonly regarded as regulatory factors in gene expression and a target in many regulatory pathways. The writers, erasers, and readers of m6A often interact with each other. In cancer research, METTL3 may regulate the protein stability of WTAP, and the upregulation of WTAP protein has carcinogenic effects only in the presence of METTL3 [99]. The m6A-related proteins involved in different types of cancer are usually regulated by multiple m6A protein factors. Due to our limited knowledge of m6A, there is still a need for extensive research on the interactions between pathways and the synergistic effects of m6A in regulating gene expression [100]. To date, various writers have been discovered, but only two erasers, FTO and ALKBH5, have been identified. Abnormal expression of FTO or ALKBH5 only leads to subtle changes in the overall level of m6A modifications in organisms, suggesting the existence of unknown demethylases in the body [6]. Thus, the identification of new m6A-related enzymes still requires in-depth research in the field of life sciences. As an important RNA epigenetic modification, further research is needed to understand how m6A interacts with DNA methylation and histone modifications to regulate gene expression, as well as the potential connections between m6A modification and other RNA modifications.

In summary, we have reviewed the current state of knowledge of the regulatory mechanisms of m6A methylation modifications in economically important traits of livestock. Available data and future work on this epigenetic mechanism will assist researchers in livestock studies to effectively manipulate the m6A modification levels of RNA using efficient methods. This could aid in developing studies to regulate the expression of key genes in biologically important functions. New molecular markers or targets for targeted breeding also can be generated with this knowledge. Together, this information may serve as a reference for genetic improvement of economic traits in animals. This is of significant importance for enhancing the production yield and quality of meat, eggs, and milk in livestock.

## Figures and Tables

**Table 1:** Main m6A-methylation-related enzymes in livestock.

Type	m6A Regulator	Cellular Localization	Function	Species	Reference
m6A writers	METTL3	Nucleus	Catalyzes m6A modification	pig, sheep, cattle, chicken,	[31,67,68,69]
	METTL14	Nucleus	Assists METTL3 to recognize the subtract	pig, sheep, cattle, chicken, duck	[31,68,70,71,72]
	METTL16	Nucleus	Catalyzes m6A modification	pig	[73]
	WTAP	Nucleus	Promotes METTL3-METTL14 heterodimer to the nuclear speckle	pig, cattle, chicken, duck	[70,71,74,75]
m6A erasers	FTO	Nucleus and Cytoplasm	Removes m6A modification	pig, cattle, sheep, chicken, duck	[76,77,78,79,80]
	ALKBH5	Nucleus	Removes m6A modification	pig, cattle, chicken	[74,81,82]
m6A readers	YTHDC1	Nucleus	Promotes RNA splicing and translocation		
	YTHDF1	Cytoplasm	Promotes mRNA translation	pig, cattle, sheep, chicken	[83,84,85,86]
	YTHDF2	Cytoplasm	Reduces mRNA stability	pig, cattle, sheep, chicken	[84,85,86,87]
	YTHDF3	Cytoplasm	Mediates the translation or degradation		
	YTHDC2	Cytoplasm	Enhances the translation of target RNA		
	IGF2BP1/2/3	Nucleus and Cytoplasm	Enhances mRNA stability	pig, sheep, chicken, duck, goose	[27,44,88,89,90]

## Data Availability

Not applicable.
